# Editorial: Translational and clinical chronobiology

**DOI:** 10.3389/fphys.2023.1191580

**Published:** 2023-03-29

**Authors:** Elise R. Facer-Childs, Minjee Kim, Sara Montagnese

**Affiliations:** ^1^ Turner Institute for Brain and Mental Health, Monash University, Melbourne, VIC, Australia; ^2^ Sleep and Circadian Rhythms Program, School of Psychological Sciences, Monash University, Melbourne, VIC, Australia; ^3^ Danny Frawley Centre for Health and Wellbeing, Melbourne, VIC, Australia; ^4^ Department of Neurology, Northwestern University Feinberg School of Medicine, Chicago, IL, United States; ^5^ Center for Circadian and Sleep Medicine, Northwestern University Feinberg School of Medicine, Chicago, IL, United States; ^6^ Center for Applied Health Research on Aging, Northwestern University Feinberg School of Medicine, Chicago, IL, United States; ^7^ Department of Medicine, University of Padova, Padova, Italy; ^8^ Chronobiology Section, Faculty of Health and Medical Sciences, University of Surrey, Guildford, United Kingdom

**Keywords:** circadian rhythms, sleep, chronotherapy, performance, neurodegeneration, light

Since the award of the 2017 Nobel prize in Physiology or Medicine to Jeffrey C. Hall, Michael Rosbash, and Michael W. Young for their discoveries of molecular mechanisms controlling the circadian rhythm (Nobelprize, 2017), the field of translational and clinical chronobiology has gained considerable interest, often featured in high-impact review articles and editorials. Along the same lines, the use of circadian indices to assess and categorise healthy and diseased individuals has opened up a new line of inquiry in various settings, ranging from clinical interventions to public health policies. However, incorporating circadian measures can be challenging and costly, especially in clinical settings, and requires close collaboration across the key stakeholders, including chronobiologists, physiologists, and clinicians (Klerman et al., 2022). We have therefore been especially pleased to have the opportunity to guest-edit the Research Topic “*Translational and Clinical Chronobiology*,” which includes six original manuscripts.

Mascaro et al. investigated the relationships between “cognitive fitness” (i.e., measures of self-control, intolerance of uncertainty, and impulsivity), mental health, and self-reported sleep measures among 82 young athletes during the COVID-19 pandemic. Women, representing approximately half of the sample studied, reported poorer cognitive fitness and mental health compared to men. Certain cognitive fitness factors, including self-control, were inversely associated with depression and anxiety, whereas others, such as premeditation, were associated with a longer sleep duration and anxiety.

Leota et al. examined the effects of travel-related jet lag on the athletic performance among professional basketball players from 11,481 the National Basketball Association games across ten seasons between 2011 and 2021. Eastward travel was associated with impaired performance for home but not for away games, after adjusting for relevant covariates. This study illustrates the far-reaching relevance of circadian physiology and potential implications of circadian disruption and translational chronobiology in real-world settings, including high level sports.

Heacock et al. utilized a large retrospective dataset collected from wrist-worn devices and linked mobile applications to examine changes in sleep-wake measures and self-reported alcohol consumption during US public holidays and Daylight Savings Time transitions. In the sample primarily comprised of men in their late 30s during the first year of the COVID-19 pandemic, significant differences were observed in sleep (increased duration, decreased consistency, and later sleep onset and offset) and alcohol consumption (a higher point prevalence) during the majority of US public holidays and the preceding nights compared to the baseline.

Zhang et al. also utilized data from wearable devices, as well as from clinical Holter monitors (i.e., medical devices that are utilized in the clinical setting to obtain 24-h ECG recordings), to examine diurnal variations in heart rate among 211 volunteers. They identified diurnal patterns predictive of cardiovascular disease risks, with a high correlation between the measures derived from wearable devices vs Holter monitors in a subset of participants. With increasing use of wearable devices and advancement in big data analytic techniques, we anticipate an increasing use of circadian-based risk stratification for early detection of risk factors and timely interventions to improve clinical outcomes.

As an example of chronotherapeutic approach, Benedetti et al. examined the effects of total sleep deprivation and timed light therapy on circulating markers of inflammation among 33 healthy volunteers and 26 patients with bipolar disorder hospitalized for an ongoing, major depressive episode. Chronotherapy was associated with significant changes in the ratio of circulating levels of interleukin-1β and its receptor antagonist (IL-1ra) and corresponding clinical response among patients, suggesting the mechanism of action through reducing inflammation.

Finally, Cremascoli et al. conducted a single-blinded, randomised controlled trial to investigate the effects of a light therapy treatment tailored to the individual circadian phase (as assessed by the dim light melatonin onset; DLMO) among 13 patients with mild-to-moderate Alzheimer’s disease. The treatment group (N = 8) demonstrated a circadian phase shift, with significant shortening of the phase angle DLMO-falling asleep time, and improvement in subjective sleep quality and cognitive performance. No change in objectively measured sleep or rest-activity variables in response to light therapy was observed.

Overall, we believe this Research Topic highlights the widespread utility and potential applicability of circadian measures and chronotherapeutic strategies, ranging from athletic performance, disease risk stratification, and personalized treatments of diseases, to public health and policy implications. These manuscripts should inspire the readers to consider relevant chronobiological constructs and incorporate them into future clinical and translational research, and encourage stakeholders to invest in increasing the understanding of how circadian factors affect health and performance.
